# Chromosome 22q11.2 Deletion Syndrome Presenting as Adult Onset Hypoparathyroidism: Clues to Diagnosis from Dysmorphic Facial Features

**DOI:** 10.1155/2013/802793

**Published:** 2013-04-30

**Authors:** Sira Korpaisarn, Objoon Trachoo, Chutintorn Sriphrapradang

**Affiliations:** Department of Medicine, Faculty of Medicine, Ramathibodi Hospital, Mahidol University, Bangkok 10400, Thailand

## Abstract

We report a 26-year-old Thai man who presented with hypoparathyroidism in adulthood. He had no history of cardiac disease and recurrent infection. His subtle dysmorphic facial features and mild intellectual impairment were suspected for chromosome 22q11.2 deletion syndrome. The diagnosis was confirmed by fluorescence in situ hybridization, which found microdeletion in 22q11.2 region. The characteristic facial appearance can lead to clinical suspicion of this syndrome. The case report emphasizes that this syndrome is not uncommon and presents as a remarkable variability in the severity and extent of expression. Accurate diagnosis is important for genetic counseling and long-term health supervision by multidisciplinary team.

## 1. Introduction

 Hypoparathyroidism leads to hypocalcemia, which can manifest with paresthesias of perioral area and extremities, tetany, muscle cramps, and carpopedal spasm. In the setting of acute severe hypocalcemia, it can present as life-threatening condition such as seizure, laryngospasm, and cardiac arrhythmia. Also patients with chronic hypocalcemia may develop neurological complications including basal ganglion calcifications and extrapyramidal neurologic manifestations [1]. 

The most common cause of hypoparathyroidism in adult is postsurgical hypoparathyroidism. Although rare genetic disorders, such as genetic mutations that involve in defective synthesis of parathyroid hormone (PTH), or abnormal parathyroid gland development or dysgenesis of thyroid glands associated with other developmental abnormalities, such as thymic hypoplasia, defects in the cardiac outflow tract, typically manifest in the neonatal or childhood period [1, 2], it is rarely reported as the etiologies of adult onset hypoparathyroidism [3–5]. These syndromes can be under-recognized due to either the unfamiliarity of physicians with the syndrome or the variable expressivity and subtle phenotype.

We report a 26-year-old man who presented with symptomatic hypocalcemia from chromosome 22q11.2 deletion syndrome, the most common microdeletion syndrome. The diagnosis could be easily missed because of subtle dysmorphic facial features.

## 2. Case Report

 A 26-year-old Thai man without underlying disease came to emergency unit because of carpopedal spasm for 4 hours. He also reported numbness and tingling at perioral area and muscle cramps in both legs. The patient's history of birth and development was normal, and he had been previously healthy. He had no history of recurrent infection or cardiac disease. He had attended 6 years of elementary school, and is working as an unskilled laborer. He did not drink alcohol or use illicit drugs. There was unremarkable family history.

 On examination, the patient was alert. His height was 153 cm (<3rd centile) and his weight was 65 kg (body mass index =27.77 kg/m^2^). His blood pressure was 100/60 mm Hg and pulse was 80 beats per minute. He had bilaterally carpopedal spasm and positive Chvostek's sign. Cardiac, chest, and abdominal examinations were unremarkable. He had no subcapsular cataract. Laboratory investigations showed hypocalcemia with calcium level of 1.5 mmol/L (normal range, 2.2–2.53 mmol/L), ionized calcium level of 0.7 mmol/L (normal range, 1.09–1.3 mmol/L), and hyperphosphatemia with phosphate level of 1.68 mmol/L (normal range, 1.07–1.49 mmol/L). Serum PTH level of 24.2 pg/mL (normal range, 15–65 pg/mL) was inappropriately normal during severe hypocalcemia. Serum magnesium and creatinine level were normal. Complete blood count and other biochemistry tests were also within normal limit. According to laboratory findings, hypocalcemia secondary to hypoparathyroidism was diagnosed. His electrocardiogram revealed prolonged QT interval with corrected QT interval of 473 msec (normal < 450 msec in male) and no ST-T change. Intravenous infusion of 10% calcium gluconate with bolus dose and continuous rate of 1 mg of elemental calcium/kg/hr was administered with concomitant oral calcium and vitamin D analogues.

**Figure 1:**
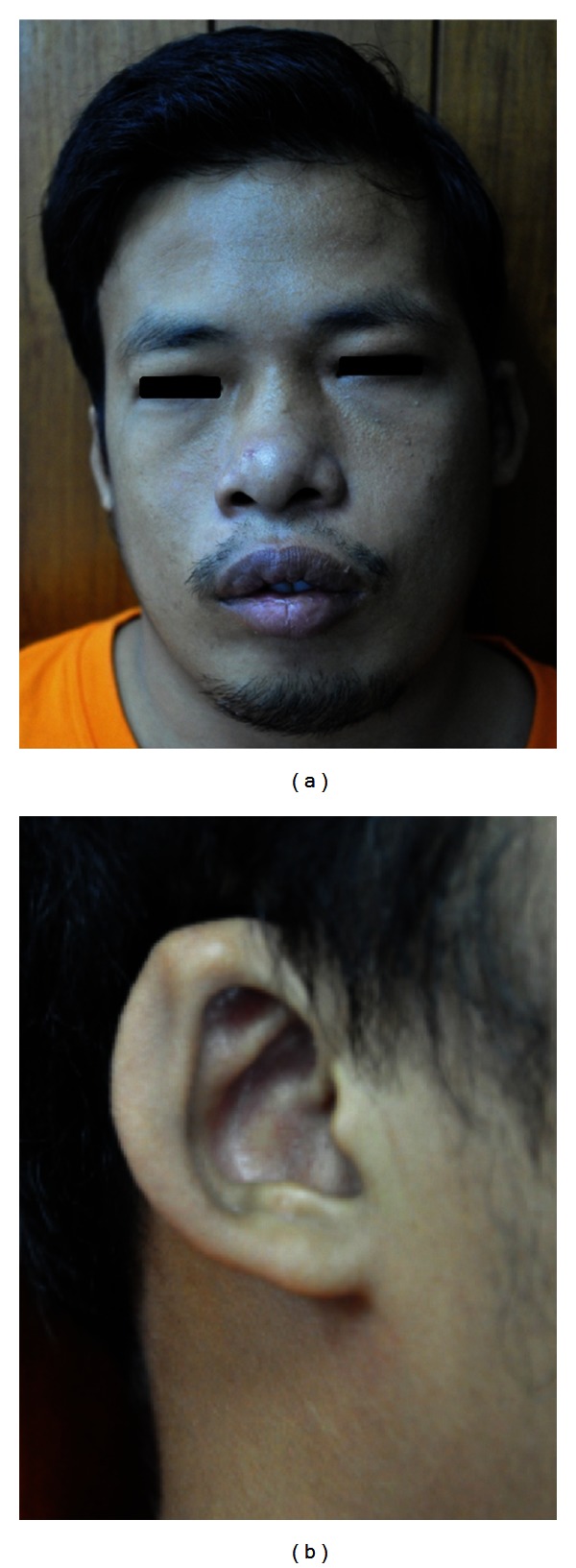
Mild dysmorphic facial features of this patient including (a) prominent forehead, hypertelorism, hooded eyelids, broad nasal bridge and prominent lips, (b) ear microtia, low set ear, thick and overfolded ear helix.

 To search for the cause of hypoparathyroidism, he had no history of neck surgery or irradiation. Morning serum cortisol level, thyroid function test, and hemoglobin A1C were normal, polyglandular autoimmune syndrome is less likely. At first presentation, we did not recognize any dysmorphic features. However, minor facial abnormalities were detected by meticulous examination. He had a prominent forehead, hypertelorism, hooded eyelids, ear microtia, low set ear, thick and overfolded ear helix, left external auditory canal stenosis, broad nasal bridge, and prominent lips (Figure 1). His intelligence quotient or IQ by Wechscler Adult Intelligence Scale was 69, which categorized as mild mental retardation. Primary hypoparathyroidism along with dysmorphic face and mild mental retardation lead to suspicion of chromosome 22q11.2 deletion syndrome in this patient. Conventional chromosome analysis using the standard G band procedure showed a normal male karyotype. Subsequently, Array Comparative Genomic Hybridization (CGH) analysis was performed using Agilent custom-designed 44 K oligonucleotide array. The array contains more than 41,000 oligonucleotide probes and has both genome-wide coverage and dense coverage in regions of known microdeletion/duplication syndromes, subtelomeric and pericentromeric regions (Genzyme Genetics, Monrovia, CA). The CGH array showed a copy loss of 192 oligonucleotide probes in the region of 22q11.2 (genomic coordinates 17289032–19687983), with an estimated size of approximately 2.40–3.14 Mb at 22q11.21 region. The 22q11.2 deletion was confirmed by a fluorescent in situ hybridization (FISH), using the HIRA probe (Abbott Molecular). The deleted region contains approximately 34 OMIM genes. Further investigations included transthoracic echocardiography, renal ultrasonography, and dental examination were all normal. Audiography showed bilateral conductive hearing loss. CT brain found bilateral basal ganglion calcification. Finally, he was diagnosed with chromosome 22q11.2 deletion syndrome (microdeletion). Genetic counseling was informed to him and his family. He was discharged from the hospital with 1,000 mg elemental calcium as calcium carbonate and 0.25 *μ*g alfacalcidiol twice daily. The serum calcium level was maintained in lower normal range to prevent hypercalciuria. During subsequent follow-up, the patient did not have any recurrence of hypocalcemic symptom.

## 3. Discusssion

 Clinically under-recognized, 22q11.2 deletion syndrome is the most common microdeletion syndrome (MIM #188400/#192430), with an estimated prevalence of 1 in 4,000 live births [6–8]. However, the actual occurrence may be higher because of variable in the severity and expressivity [9, 10]. Patients with 22q11.2 deletion tremendously display wide spectrum of manifestations include cardiac anomalies (conotruncal abnormalities for example, tetralogy of Fallot, type B interrupted aortic arch, truncus arteriosus, right aortic arch, and aberrant right subclavian artery), immune deficiency from thymic hypoplasia, behavioral and cognitive disorders, psychiatric disorders (schizophrenia and major depressive illness), mental retardation, hypoparathyroidism, renal anomaly, central nervous system anomaly, palatal defects, and facial dysmorphic feature [11, 12]. The diverse presentations are described as many syndromes, for example, DiGeorge syndrome, velocardiofacial syndrome, conotruncal anomaly face syndrome, autosomal dominant Opitz G/BBB syndrome, and Cayler cardiofacial syndrome [13]. Although this list of syndromes may appear quite confusing, it is understandable because the diagnoses were formerly described by clinicians concentrating on their particular areas of interest. After the widespread use of FISH analysis, patients with a deletion became collectively referred to by their chromosomal etiology: the 22q11.2 deletion syndrome. 

Hypoparathyroidism due to parathyroid aplasia or hypoplasia is found over a half of affected individuals with 22q11.2 deletion [10]. Although this condition is usually diagnosed in neonatal period due to an abrupt interruption of maternal transport of calcium [14, 15], it has been described to be late onset because of adaptation to hypocalcemia by developing parathyroid glands hypertrophy [3–5]. PTH secretion may be adequate to sustain normocalcemia under basal conditions but cannot effectively increase in response during the time of stress such as cardiac surgery, illness, puberty, or pregnancy [16]. Even though our patient did not have precipitating factors. Chromosome 22q11.2 deletion syndrome is often under-recognized because of subtle features. This case report illustrates that careful physical examinations can lead physicians to the correct diagnosis. In this patient, dysmorphic facial anomalies, short stature, and intellectual impairment raised our suspicion for this diagnosis. The common facial anomalies in this syndrome include abnormal nasal shape (bulbous nasal tip, tubular nose), auricular defects (round shape, microtia, low set, posteriorly rotated ear and deficient in the vertical diameter with abnormal folding of the pinna), hooded eyelids, telecanthus, upward or downward slanting eyes, hypernasal speech associated with submucous or overt palatal clefting, narrow/elongated face, micrognathia, and flattened malar prominence [11, 15, 17]. Physical signs can help physicians to avoid under-diagnosis of other genetic syndromes of hypoparathyroidism (Table 1). In addition, patients with PTH resistance who suspect for Albright hereditary osteodystrophy or pseudohypoparathyroidism type 1a typically present as round face, short stature, obesity, and short 4th metatarsals [18].

The 22q11.2 deletion is almost always too small to be identified with cytogenetic studies using standard chromosome banding techniques alone. FISH studies have allowed clinical laboratories to identify patients with 22q11.2 deletions. Within this region, the *TBX1* gene has been shown to carry inactivating point mutations in some DiGeorge patients. This gene encodes a T-box transcription factor, that is, widely expressed in those embryonal tissues that give rise to many of the organs that can be clinically affected in this syndrome [19]. Over 90% of the deletions occur *de novo* while 10% of patients have parentally inherited deletion [20, 21]. Risk of recurrence in the offspring is up to 50% in each pregnancy [22]. Genetic counseling should be performed in every patient with this deletion. 

 In conclusion, adult onset hypoparathyroidism can be a presentation of chromosome 22q11.2 deletion syndrome. Concentrating physical examination is the key to diagnosis and finally leads to appropriate evaluation and management by multidisciplinary team. 

## Figures and Tables

**Table 1:** Summary of clinical clues for diagnosis of genetic disorders associated with hypoparathyroidism other than 22q11.2 deletion syndrome.

Disorders	Associated features
Isolated hypoparathyroidism from mutations in *PTH*, *GCMB*, *CaSR* gene	None

Polyglandular autoimmune syndrome	Addison's disease, alopecia, autoimmune thyroid disease, diabetes mellitus type 1, mucocutaneous candidiasis, vitiligo

Hypoparathyroidism-retardation-dysmorphism syndrome	
(i) Sanjad-Sakati syndrome	Microcephaly, microphthalmia, mental retardation, short stature, small size of hands, feet, abnormal teeth
(ii) Kenny-Caffey syndrome	Dwarfism, eye abnormalities, medullary stenosis of the long bone

Hypoparathyroidism-deafness-renal dysplasia syndrome	Deafness, renal dysplasia

Mitochondrial disorders associated with hypoparathyroidism	
(i) Kearns-Sayre syndrome	Cardiac conduction abnormalities, ophthalmoplegia, retinal pigmentation
(ii) Mitochondrial encephalomyopathy, lactic acidosis, and stroke-like episodes (MELAS syndrome)	As the name implies
